# Valorization of fish processing by-products through combined enzymatic and microbial hydrolysis: nitrogen recovery and fertilizer efficiency in wheat

**DOI:** 10.3389/fnut.2026.1757182

**Published:** 2026-02-23

**Authors:** Tamara Solis, Silvana Valdivia, Alejandra Vergara, Marcela Carvajal, Ignacio Seguel, Pedro Valencia

**Affiliations:** 1 Programa de Doctorado en Biotecnología, Pontificia Universidad Católica de Valparaíso, Universidad Técnica Federico Santa María, Valparaíso, Chile; 2 Centro de Biotecnología Daniel Alkalay Lowitt, Universidad Técnica Federico Santa María, Valparaíso, Chile; 3 Departamento de Ingeniería en Diseño, Universidad Técnica Federico Santa María, Valparaíso, Chile; 4 Future Botanics, Santiago, Chile; 5 Escuela de Alimentos, Pontificia Universidad Católica de Valparaíso, Valparaíso, Chile

**Keywords:** biostimulant effect, byproduct valorization, fertilizers, fish byproducts, nitrogen recovery, protein hydrolysates

## Abstract

The bioconversion of fish by-products has been evidenced as a sustainable process to convert food waste into high-value products. In the present study, protein hydrolysates were produced from fish by-products by different bioprocesses and evaluated as fertilizers in wheat (*Triticum aestivum L.*) on a nitrogen-equivalent basis. Fish by-products were processed through grinding prior to bioconversion. Enzymatic hydrolysis was performed using Alcalase at 55 °C, pH 6.5, and a 3-h reaction, while microbial conversion was assessed using a lactic culture at 40 °C, pH 6.5, and a 10-day culture. Hydrolysates obtained by enzymatic and microbial bioconversion were evaluated as fertilizers by adding 30 mg after 7 and 14 days to wheat seeds sown under controlled conditions. Protease and microbial hydrolysis generated high concentrations of *α*-amino groups, yielding 100 mM and 170 mM, respectively. The combined process exhibited a synergistic effect, yielding 226 mM of *α*-amino groups and 33% of protein recovery. Plant growth assays were conducted under controlled conditions using nitrogen-equivalent doses of each hydrolysate. Microbial and combined enzymatic-microbial hydrolysates generated average plant lengths of 52 cm and 54 cm compared to 44 cm in the control, while plant biomass reached 1.7 g and 2.3 g with microbial and combined enzymatic-microbial hydrolysates compared to 0.7 g in the control. Photosynthetic parameters remained within normal physiological ranges from 2.5 to 3.3 for performance index (PI) and from 0.78 to 0.80 for maximum quantum efficiency (Fv/Fm). The integration of enzymatic and microbial catalysis produced the most effective biostimulant activity, highlighting the value of combining enzymatic specificity with microbial metabolic versatility. These findings support fish-derived protein hydrolysates as efficient and eco-friendly fertilizers that are capable of improving plant growth while contributing to sustainable and integral utilization of natural resources.

## 1 Introduction

Fertilizers derived from organic sources are increasingly recognized as key components of sustainable agricultural systems due to their ability to supply nutrients while improving soil health and reducing environmental impacts. In contrast to synthetic fertilizers, which provide rapidly available mineral nutrients but are frequently associated with soil acidification, eutrophication, and contamination by heavy metals or radionuclides, organic fertilizers release nutrients more gradually and often contain bioactive compounds that stimulate plant growth and enhance stress tolerance (1–3). Some inconsistencies in the term “biofertilizer” and “biostimulant” have been highlighted by Santos et al. (4). The definition of biofertilizer corresponds to any agent containing microorganisms that support the growth of plants by enhancing the nutrient supply, while biostimulant is any agent (substance or microorganism) applied to plants that enhance nutrition efficiency, abiotic stress tolerance, and/or crop quality traits, regardless of its nutrient content (5, 6). According to these definitions, biofertilizers are nutrition enhancers, while biostimulants are functional enhancers. We have adopted this nomenclature, and we will denominate fertilizers to the protein hydrolysates obtained from fish by-products, regardless of their enzymatic or bacterial processing. The concept of biostimulant will be used by referring to a property, not a substance or a product.

Protein hydrolysates, in particular, have attracted growing attention as fertilizers because they consist of low-molecular-weight peptides and free amino acids with high solubility and bioavailability, facilitating nutrient uptake and metabolic activation in plants (1, 7). Marine-derived protein hydrolysates have shown promising agronomic effects, such as enhanced seed germination, root development, shoot growth, and photosynthetic efficiency (1, 2, 8). Fish-processing by-products are especially attractive feedstocks for fertilizer production, as the extraction and aquaculture industries generate large volumes of residues such as heads, viscera, skin, fins, and bones, which may contain up to 20% residual muscle tissue and exhibit high protein contents ranging from 8 to 35%, depending on species and tissue type (9–12). The valorization of these residues through protein hydrolysis therefore represents a sustainable strategy to recover organic nitrogen, phosphorus, and essential micronutrients, while simultaneously reducing the environmental burden associated with waste disposal (1, 2, 8, 13).

Several methods have been developed to convert fish residues into protein hydrolysates. Enzymatic hydrolysis has emerged as a preferred method for protein recovery and transformation due to its high specificity, mild operating conditions, and ability to generate peptides with controlled molecular weight and tailored functional properties. Compared with chemical hydrolysis, enzymatic approaches impede the formation of toxic by-products and preserve essential amino acids, resulting in hydrolysates with improved solubility, digestibility, and bioavailability (14). Enzymatic hydrolysis using commercial proteases allows precise control of reaction conditions and typically results in high yields of soluble proteins and peptides (15). These characteristics are particularly relevant for the production of peptides exhibiting functional and bioactive properties (16–18). Recent experimental studies have also indicated that enzymatic hydrolysis is applicable beyond marine resources, extending to emerging protein sources, such as insects (19, 20). Despite its advantages, several challenges limit the large-scale industrial implementation of enzymatic protein hydrolysis. These challenges include the reduction of proteases cost, variability in raw material composition, and sensory issues, such as bitterness in protein hydrolysates (21). Downstream processing and purification further contribute to technological and economic constraints. Membrane filtration is commonly used to fractionate peptides by molecular weight, achieving higher concentrations of peptides with bioactive properties (14, 22). However, this technology is also associated with high capital investment, membrane fouling, energy consumption, and cleaning requirements (23). Continued advancements in enzyme technology, process integration, and biorefinery design are expected to play a decisive role in overcoming current limitations and enabling the full industrial and commercial potential of these protein-derived ingredients.

Alternatively, microbial or fermentative hydrolysis—most commonly mediated by lactic acid bacteria (LAB)—offers a cost-effective and environmentally friendly strategy based on endogenous microbial proteolytic activity, although it generally requires longer processing times than enzymatic treatments (24, 25). Fermentation of fish-processing by-products with LAB has been reported to generate protein hydrolysates with improved antioxidant and antibacterial properties (26, 27). More recently, hybrid strategies combining enzymatic and microbial hydrolysis have been proposed to improve protein solubilization efficiency, reduce processing time, and enhance the agronomic performance of the resulting fertilizers, as well as their potential benefits for soil health (1, 2, 28, 29).

The use of protein hydrolysates as plant fertilizers with possible biostimulant properties offers a unique opportunity to bypass the limitations raised from their direct application as human nutritional ingredients: sensory difficulties, clinical evaluations, and market constraints.

Despite these advancements, the current state of research reveals a relevant knowledge gap. Few studies have systematically compared enzymatic, microbial, and combined hydrolysis processes using fish residues as substrates under equivalent experimental conditions. Furthermore, a fertilizer’s performance is often evaluated on a mass basis rather than under standardized nitrogen inputs, which hampers a rigorous comparison of its true fertilizing efficiency.

To address these limitations, the present study proposes a comparative assessment of enzymatic, microbial, and combined protease–microbial treatments for the bioconversion of fish wastes into protein hydrolysates. The specific objectives were to evaluate and compare the efficiency of protein solubilization and nitrogen recovery achieved by each process and to evaluate the effects of the resulting hydrolysates on wheat plant growth and photosynthetic performance under equivalent nitrogen application rates.

It is expected that this approach will clarify the relative advantages and limitations of each bioprocessing strategy, identify potential synergistic effects in combined treatments, and provide robust evidence supporting the use of marine-derived protein hydrolysates as eco-efficient fertilizers. These results aim to contribute to the development of scalable, sustainable solutions for the valorization of fish-processing residues within a circular bioeconomy framework.

## 2 Materials and methods

### 2.1 Materials

Fish-processing residues (FR), such as heads, fins, and bones from an approximately even mixture of *Brama australis* and *Merluccius australis*, were collected in polystyrene boxes from a local fish market (Concepción, Chile). To homogenize and reduce particle size, the residues were processed using an industrial cutter (Talsa PSV C15), followed by a meat grinder (Omega TL32), and stored at −18 °C until further use. The total nitrogen content of the raw material was determined by the Kjeldahl method, yielding a value of 2.86% (w/w), equivalent to 17.9% (w/w) total protein. The initial FR moisture was 72%. Molasses was obtained from COAGRA (Los Ángeles, Chile). For enzymatic hydrolysis, Alcalase® 2.5 L (Novozymes, Bagsvaerd, Denmark) was used as the protease containing 2.4 AU/g. For microbial hydrolysis, cultures of *Lactobacillus bulgaricus* and *Streptococcus thermophilus* were employed and sourced directly from a commercial natural yogurt (Oikos, Danone). A commercial fertilizer was used as a reference. For the plant assays, commercial wheat seeds (Stender GmbH, UK) were used with blond peat (Stender GmbH, UK) and class A grape pomace compost (NORSolución, Chile) as growth substrates.

### 2.2 Protein hydrolysis

Protein hydrolysis of the FR was performed using two catalytic approaches, protease and microbial culture, along with their combination. For enzymatic hydrolysis, 200 g of the reaction mixture was placed in a 400-mL glass reactor equipped with a helical stirrer operating at 400 rpm. The reaction mixture consisted of 70% (w/w) FR and 30% (w/w) of a 50% (w/w) molasses solution. The reactor was immersed in a thermostatically controlled water bath at 55 °C under constant stirring and at native pH (6.5), in accordance with previous standardization and optimization studies (15, 30). Once the target temperature was reached, a time-zero sample was taken, the enzyme was added, and the reaction was run for 3 h. Samples were subsequently collected at 5, 10, 20, 30, 60, 90, 120, 150, and 180 min to monitor the progression of hydrolysis. Three different doses of Alcalase were evaluated: PD1 = 0.5 UA·kg^−1^ FR, PD2 = 1.0 UA·kg^−1^ FR, and PD3 = 2.0 UA·kg^−1^ FR.

For microbial hydrolysis, two setups were evaluated: one using bacterial inoculum only and another supplemented with protease dose 1 (PD1). The process was conducted at 40 °C and native pH using 100 g of reaction mixture in 500 mL Erlenmeyer flasks under orbital agitation (250 rpm). Each flask was inoculated with 10 mL of microbial culture (*L. bulgaricus* and *S. thermophilus*) and incubated for 10 days. Samples were collected at 0, 24, 48, 72, 96, 168, and 240 h for subsequent analyses.

Samples were immediately mixed with an equal volume of 10% trichloroacetic acid and centrifuged for 10 min at 10,000 x *g*. The supernatant was recovered and analyzed for the concentration of released *α*-amino (α-NH) groups. Upon completion of the total processing time, the whole reaction mixture was centrifuged under the same conditions described above. The supernatant was recovered and analyzed for protein content by the Kjeldahl method to calculate protein recovery, defined as the protein transferred from the FR to the soluble phase. All procedures were performed as described in previous publications (15, 30, 31). Control experiments were conducted under identical conditions but without catalyst addition (bacterial inoculum or protease). Each treatment was performed in duplicate.

### 2.3 Evaluation of the effect of protein hydrolysates on wheat growth

Wheat (*Triticum aestivum* L.) was cultivated from commercial seeds using 20% blond peat (Stender GmbH, UK) and 80% class A (according to Chilean standard NCh 2,880 Of. 2004) grape pomace compost as substrate (NORSolución) with the following physical and chemical compositions: pH 6.8; EC:3.0 dS/m, humidity 30%; C/N = 25:1, nitrogen 1.7%, P_2_O_5_ 1.2%, K_2_O 1.3%, and organic matter 30%. Fifty disposable 400-mL pots were each sown with 10 seeds and maintained at room temperature in an incubation chamber (23 °C with a 16/8 h photoperiod light/dark).

Pots were irrigated at 7 and 14 days post-germination with 50 mL of either water (control) or the same amount of water with 30 mg of nitrogen from the different fertilizers for 2 weeks. The tested fertilizers corresponded to hydrolysates obtained from (i) commercial reference (R), (ii) microbial hydrolysis (M), (iii) microbial + protease hydrolysis (MP), and (iv) enzymatic proteolysis (P) with a dose of 2.0 UA·kg^−1^ FR. The applied fertilizer doses were standardized to deliver 30 mg of nitrogen per pot, ensuring nitrogen-equivalent treatments.

After 14 and 21 days of cultivation, 10 plants from each treatment were sampled to determine the following parameters: shoot length, root length, shoot biomass, root biomass, photosynthetic performance index (PI), and maximum quantum efficiency (Fv/Fm). PI and Fv/Fm measures were obtained from data collected using a Pocket PEA chlorophyll fluorimeter (Hansatech Instruments, Norfolk, England) after adapting the sample to darkness for 30 min before each measurement. Shoot and root lengths were measured with a ruler across the vertical dimension, and their masses were determined using an analytical balance.

### 2.4 Analysis and statistics

Free *α*-NH groups were quantified using the *o*-phthaldialdehyde (OPA) method with serine as the standard (32). Total nitrogen in both the FR and the soluble phase was determined using the Kjeldahl method. The soluble phase was obtained by centrifugation of the reaction mixture. The mass percentage of the soluble phase was calculated relative to the total mass of the reaction mixture. Protein extraction was calculated as the proportion of total protein transferred from the FR to the soluble phase. The degree of hydrolysis (DH) was determined from the ratio between released *α*-NH groups and the total peptide bonds, which, in turn, was calculated from the difference between total nitrogen and initial free α-NH groups (15, 30). The productivity of the hydrolysis process was calculated as the protein mass generated in the soluble phase per processing time.

Experimental data were subjected to the analysis of variance (ANOVA) to determine significant differences among treatments. The test statistic *F*_0_ for the null hypothesis of equal means was compared with *F_α,a-1, N-a_*, where *α* is 0.05, *a* is the number of treatments, and *N* is the number of experiments. Pairwise mean comparisons were performed using Tukey’s test, where the difference between two means was evaluated according to: $T_{\alpha }=q_{\alpha }(a,N-a)\sqrt{MS_{E}/n}$ , where *MS_E_* is the mean square error and *n* is the number of replicates. All statistical tests were considered significant at a *p-*value of < 0.05.

## 3 Results and discussion

### 3.1 Enzymatic and microbial hydrolysis kinetics

**Figure 1:**
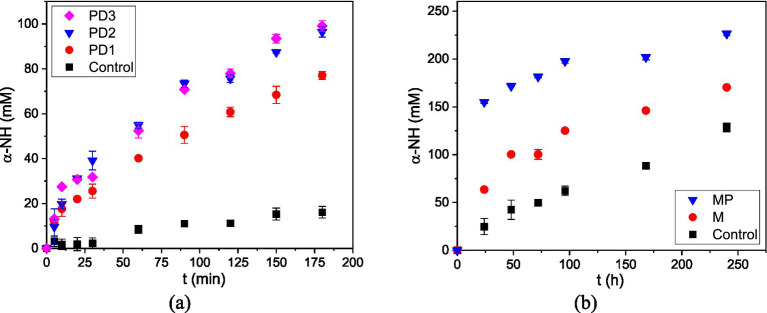
Release of *α*-NH groups during the hydrolysis of FR proteins at different treatments: **(a)** Enzymatic hydrolysis at different Alcalase doses (PD, protease dose) and **(b)** Microbial culture (M, microbial inoculum; MP, microbial inoculum + protease). Each value was obtained from two hydrolysis experiments (*n* = 2).

Peptide bonds in fish-residue proteins were cleaved during hydrolysis, releasing *α*-NH groups into the soluble phase. As shown in Figure 1a, the enzymatic hydrolysis of FR with increasing protease doses exhibited a clear dose-dependent pattern. The addition of PD3 did not generate a further increase in released *α*-NH groups compared to PD2. This protease-dose saturation effect was observed in previous studies (15, 30).

In Figure 1b, corresponding to microbial hydrolysis, a progressive increase in *α*-NH groups was also observed throughout the reaction period. The treatment combining microbial inoculum and protease (MP) resulted in higher α-NH concentrations than the microbial treatment (M) and the control (C), indicating a synergistic interaction between enzymatic and fermentative activity. These findings are consistent with previous reports, highlighting the cooperative effect of microbial and exogenous proteases in accelerating peptide formation (25, 27, 28). The DH was calculated for the final samples of each treatment, i.e., 3 h for the enzymatic treatment and 10 days for microbial treatment. The results are shown in Table 1.

**Table 1:** Degree of hydrolysis obtained for each treatment.

Treatment	DH (%)
Control microbial (CM)	3.79
Microbial (M)	5.61
Microbial + Protease (MP)	8.53
Control protease (CP)	0.46
Protease dose 1 (PD1)	1.89
Protease dose 2 (PD2)	2.90
Protease dose 3 (PD3)	3.09

The DH corresponds to the cleavage of peptide bonds with regard to the total number of peptide bonds in the original proteins. The results showed higher hydrolysis in all microbial treatments compared to proteolytic treatments. The main factor affecting these values was the processing time. As mentioned in procedures, the microbial treatment lasted for 10 days compared to 3 h for proteolytic treatment. Even the microbial control experiment produced a higher DH than that observed with the highest protease dose (PD3). Previous studies on the hydrolysis of salmon frame proteins reported DH values between 2 and 5% under similar operating conditions (30). The DH observed in the microbial control experiment can be explained by the presence of native microbiota in the FR. However, inoculation with LAB effectively increased the number of active bacteria, which exhibited significant proteolytic activity, and led to higher DH during the bioconversion of FR proteins. These findings indicate that inoculation with LAB is an effective treatment to achieve high DH in FR proteins.

### 3.2 Soluble phase and protein solubilization

**Figure 2:**
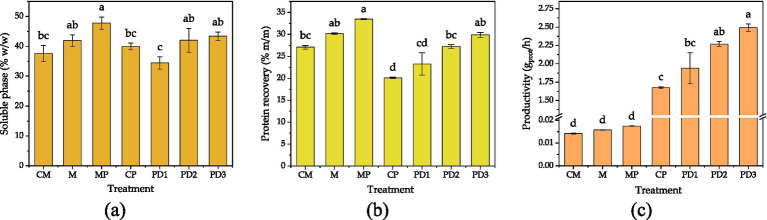
Production parameters after the hydrolysis of FR proteins for different treatments: **(a)** Soluble phase, **(b)** protein recovery, and **(c)** protein productivity. CM, Microbial control; M, microbial; MP, microbial + protease; CP, protease control; PD, protease doses. Captions indicate significant differences between treatments. Each value was obtained from two hydrolysis experiments (*n* = 2).

During hydrolysis, FR proteins were solubilized, increasing their concentration in the aqueous phase of the reaction mixture. The proportion of the soluble phase relative to the total reaction mixture is presented in Figure 2a, with values ranging from 36 to 48%. A general trend of increasing solubilization was observed with higher catalyst doses. This trend was particularly marked in microbial treatments, where both inoculum and protease addition enhanced the percentage of the soluble phase obtained. The soluble phase contained hydrolyzed proteins of lower molecular weight and higher hydrophilicity, which favored their dissolution in water.

Protein recovery, calculated regarding the total protein content in FR, correlated positively with the catalyst dose, as shown in Figure 2b. The extraction values typically ranged from 20 to 33% (w/w), as indicated in previous studies (15, 30). In both enzymatic and microbial systems, higher catalyst concentration led to increased protein extraction. The combined microbial inoculum and protease yielded the highest extraction, while in enzymatic hydrolysis, increasing protease dosage produced a proportional increase in solubilized protein.

The protein productivity of each treatment, expressed as the mass of soluble protein produced per time, is shown in Figure 2c. A positive correlation between catalyst dose and productivity was observed in both systems. Productivity values differed by two orders of magnitude between microbial and enzymatic treatments. These results are a consequence of the inherent time for both enzymatic and microbial processes. Enzymatic and microbial treatments were conducted for 3 h and 10 days, reaching approximately 100 mM and 200 mM of *α*-NH, respectively. With microbial treatment alone, 100 mM of α-NH can be achieved in 4 days, whereas the combined enzymatic-microbial process reaches this level in less than 1 day. Thus, the process duration can be chosen according to the requirements of the final product. In this context, the biostimulant effect can be evaluated as a function of fermentation time, which could be explored in future studies.

Considering that both enzymatic and microbial treatments achieved approximately 30% protein recovery, it can be inferred that microbial treatment reached a higher degree of hydrolysis (higher release of *α*-NH groups for the same amount of soluble protein). As presented in Table 1, microbial treatments exhibited higher DH values than protease-only treatments, indicating that LAB-excreted proteases contributed to enhanced protein hydrolysis.

### 3.3 Plant growth response

The agronomic potential of the hydrolysates was evaluated using wheat plants (*Triticum aestivum* L.) fertilized with different protein hydrolysates. Growth parameters, such as shoot and root length, shoot and root biomass, photosynthetic performance index (PI), and maximum quantum efficiency (Fv/Fm), were quantified after 14 and 21 days of cultivation. Plants were fertilized with 30 mg of nitrogen from protein hydrolysates prepared through protease, microbial, and microbial–proteolytic treatments. The nitrogen content of fertilizers used is shown in Table 2.

**Table 2:** Nitrogen content of fertilizers used in plant growth assays.

Fertilizer	Nitrogen content (% w/w) (value ± se)
Commercial reference (R)	3.00*
Microbial (M)	1.59 ± 0.034
Microbial + Protease (MP)	1.65 ± 0.050
Protease (P)	1.53 ± 0.001

*Data from product labeling.

**Figure 3:**
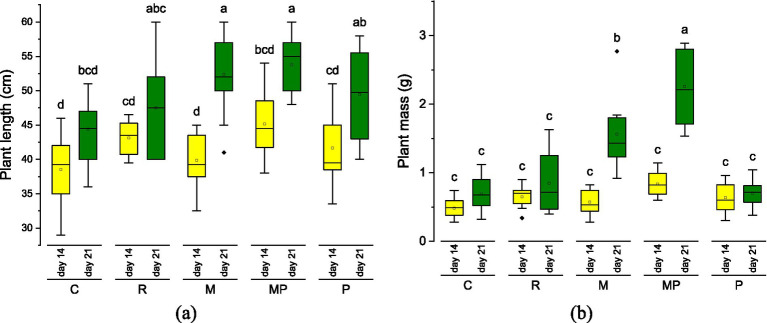
Plant growth parameters for different fertilizers: **(a)** Plant length and **(b)** plant mass after 14 days (yellow) and 21 days (green) of growth. Treatment labels are C, control; R, reference; M, microbial; MP, microbial + protease; P, protease. Each mean value was obtained from 10 wheat plants (*n* = 10).

The results for plant length and mass are shown in Figure 3. The control (water irrigation) exhibited the lowest growth. Treatments with microbial hydrolysate (M) and microbial–protease hydrolysate (MP) produced significantly higher growth, considering both length and mass after 14 and 21 cultivation days.

All treatments, including the control, showed a significant increase in plant length at 21 days of cultivation compared with the previous measure at 14 days. Plant mass also increased significantly in the control and in plants treated with microbial (M) and microbial + protease (MP) fertilizers at 21 days compared to 14 days of cultivation. Detailed data for shoot and root parameters are presented in Table 3. A significant increase in shoot length was observed between 14 and 21 days of cultivation, whereas no significant increase in root length was observed during the same period. The lack of growth in root length was likely due to the limited pot volume, which may explain why growth was primarily observed in the shoot. However, root mass increase was significant just for the microbial (M) and microbial + protease (MP) treatments. These findings evidenced that hydrolysates produced by combined hydrolysis treatment improved plant growth efficiency due to an increased availability of nitrogenous and bioactive compounds. The superior and statistically significant performance of the microbial–protease hydrolysate suggests a synergistic effect between enzymatically generated peptides and microbial metabolites, similar to that reported in soil–plant systems using hydrolysates from marine by-products (28).

**Table 3:** Growth parameters and standard error (SE) are detailed for shoot and root during the cultivation of wheat plants with different fertilizer additions (*n* = 10).

Treatment	Shoot length (cm) (value ± se)		
	Parameter day 14	Parameter day 21	Significant difference
C	22.62 ± 1.25	28.40 ± 1.18	Yes
R	26.71 ± 0.66	30.30 ± 1.63	Yes
M	23.75 ± 0.77	36.40 ± 1.29	Yes
MP	26.75 ± 0.54	36.44 ± 0.91	Yes
P	23.78 ± 1.24	32.88 ± 0.99	Yes

Treatment	Root length (cm) (value ± se)		
	Parameter day 14	Parameter day 21	Significant difference
C	15.92 ± 0.51	16.00 ± 1.03	No
R	16.42 ± 0.52	17.2 ± 0.66	No
M	16.12 ± 0.67	16.00 ± 1.07	No
MP	18.42 ± 1.17	17.33 ± 0.69	No
P	17.89 ± 0.73	16.59 ± 0.99	No

Treatment	Shoot mass (g) (value ± se)		
	Parameter day 14	Parameter day 21	Significant difference
C	0.305 ± 0.022	0.473 ± 0.045	Yes
R	0.473 ± 0.031	0.550 ± 0.066	No
M	0.392 ± 0.029	0.963 ± 0.081	Yes
MP	0.595 ± 0.044	1.397 ± 0.152	Yes
P	0.451 ± 0.055	0.564 ± 0.032	No

Treatment	Root mass (g) (value ± se)		
	Parameter day 14	Parameter day 21	Significant difference
C	0.177 ± 0.024	0.215 ± 0.034	No
R	0.173 ± 0.019	0.295 ± 0.075	No
M	0.180 ± 0.025	0.598 ± 0.103	Yes
MP	0.238 ± 0.027	0.861 ± 0.057	Yes
P	0.184 ± 0.026	0.136 ± 0.018	No

C, control; R, reference; M, microbial; MP, microbial + protease; P, protease.

Recent reports confirm that protein hydrolysates promote plant growth not only through nitrogen supply but also through peptide-mediated signaling and improved nitrogen assimilation efficiency (1, 29). The study of Mironenko et al. (13) is the most similar published study to compare the present results. They used commercial liquid protein hydrolysate from Rainbow trout (*Oncorhynchus mykiss*) to evaluate the effect on wheat growth and yield. Thirty wheat seeds were sown in pots containing 600 g of soil mixed with different amounts of dried hydrolysate, ranging from 0.2 g to 1.2 g of nitrogen. In our study, 10 wheat seeds were sown in pots containing approximately 300 g of soil, which were fertilized on days 7 and 14. Mironenko et al. reported that wheat plants grew between 18 cm and 25 after 14 days, whereas, in our study, wheat plants reached between 44 and 54 cm after 21 days, following the addition of 30 mg of nitrogen on the 7th and 14th day of cultivation. The main difference between the two experimental designs lies in the organic nature of the growth substrate used in our study compared to the soil used by Mironenko et al., which was composed of quartz sand (70%), silt (23%), and clay (7%). The organic nature of the growth substrate also explains why there was no significant difference in wheat growth between the enzymatic hydrolysate and the control. The enzymatic hydrolysate may not have provided a significantly higher nitrogen input compared to the control. Additional experiments are required to discriminate against the potential factors explaining the lack of a significant effect of the enzymatic protein hydrolysate: substrate richness, lower DH, and the absence of microbial activity. On the other hand, significant differences were observed between microbial treatments and the control, which can be explained by the contribution of LAB and its metabolites to the growth of wheat plants. In turn, Pasković et al. (1) described similar stimulation trends with low molecular weight peptides. Compared to the anchovy-derived hydrolysates reported by Tütüncü (2), the results presented in this research show comparable or superior plant growth with equivalent doses of nitrogen, highlighting the efficiency of the combined enzymatic–microbial process.

As mentioned by Colla et al. (29), there are direct and indirect biostimulant effects. The protein hydrolysates directly influence plant physiology by stimulating metabolic pathways due to the contribution of nitrogen molecules needed for biomass production. The indirect mechanisms involve an enhancement of nutrient availability and the stimulation of the plant microbiome. We can infer that the indirect mechanism mostly contributed to the observed results by providing LAB and their excreted metabolites, thus improving soil health. This also would explain why the commercial fertilizer (R) did not generate a significant difference in wheat growth compared to the control and the hydrolysate from protease treatment. It is possible that the commercial fertilizer was not microbiologically active when used in these experiments. We can also infer that a higher DH and the presence of microbial culture in the fertilizer were the main factors enhancing the stimulation of plant growth. Future studies are required to independently elucidate the contribution of each factor.

### 3.4 Photosynthetic performance

**Figure 4:**
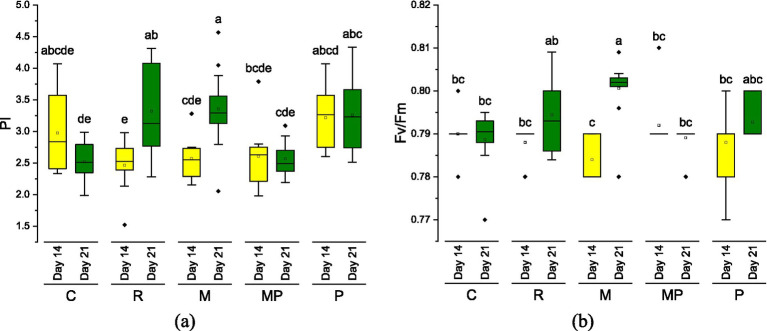
Photosynthetic performance of plants after 14 days (yellow) and 21 days (green) of cultivation. **(a)** Photosynthetic performance index (PI) and **(b)** maximum quantum efficiency. Treatments labels are C, control; R, reference; M, microbial; MP, microbial + protease; P, protease. Each mean value was obtained from 10 wheat plants (*n* = 10).

Photosynthetic indicators (Figure 4) revealed moderate variations among treatments for PI and Fv/Fm. Both the commercial and experimental hydrolysates resulted in slightly lower PI values than the control; however, Fv/Fm remained stable, indicating no photochemical damage to photosystem II (PSII). This pattern of reduced PI with stable Fv/Fm is well established in plant physiology (33), suggesting that PSII integrity remains intact while temporary limitations occur in electron transport or biochemical energy use. Interestingly, both parameters PI and Fv/Fm displayed significant differences during plant growth for the reference and microbial fertilizers, as observed in Figures 4A,B. Despite these differences in photosynthetic performance, we can infer that these values do not represent a practical difference based on the plant growth observed.

In the present study, although some treatments resulted in a reduction of the photosynthetic performance index (PI), the maximum quantum efficiency of photosystem II (Fv/Fm) remained statistically unchanged compared to the control. The stability of Fv/Fm values indicates the absence of photochemical damage to PSII, confirming that the primary photochemical reactions were not impaired. This response pattern—characterized by reduced PI while maintaining stable Fv/Fm—is well-documented in plant physiology and is typically associated with functional or metabolic limitations downstream of PSII rather than structural damage to the photosynthetic apparatus (34, 35). The stable Fv/Fm values observed in this study in plants are consistent with the findings of Xu and Mou (8), confirming the absence of photochemical damage to PSII. Similar responses have been reported in crops treated with protein hydrolysates derived from fish, plant, and animal residues. Ertani et al. (36) observed transient reductions in photosynthetic performance parameters in maize following protein hydrolysate application, while Fv/Fm values remained unaffected, suggesting short-term metabolic adjustments rather than stress-induced photoinhibition. Comparable findings were reported by Colla et al. (29) in tomato plants, in which organic fertilizers enhanced biomass accumulation despite moderate variations in chlorophyll fluorescence indices.

Several physiological mechanisms may explain this behavior. First, transient stomatal or metabolic limitations may occur after the application of organic fertilizers. Protein hydrolysates can influence osmotic balance, stomatal conductance, and water uptake, temporarily restricting CO₂ assimilation without compromising PSII integrity (37, 38). Second, the high availability of organic nitrogen, particularly in the form of amino acids and peptides, may induce a redistribution of metabolic resources toward protein synthesis, cell division, and growth, thereby reducing the instantaneous demand for photosynthetic electron transport and carbon fixation (36, 39). Third, bioactive compounds and physicochemical properties of the hydrolysates, such as pH and electrical conductivity, may modulate enzymatic activity, hormonal signaling, and stomatal behavior, affecting photosynthetic efficiency at the functional rather than at the photochemical level (29, 40, 41).

Overall, the maintenance of Fv/Fm values across treatments indicates that protein hydrolysates did not exert toxic effects on the photosynthetic machinery. Instead, the observed variations in PI are consistent with adaptive physiological responses previously reported for plants supplied with organic fertilizers. These findings reinforce the concept that protein hydrolysates primarily function as metabolic regulators rather than stress-inducing agents, thereby promoting growth while preserving the structural and functional integrity of the photosynthetic system.

## 4 Conclusion

This study evidenced that enzymatic hydrolysis, microbial bioconversion, and their combined applications constitute effective and complementary strategies for transforming fish-processing residues into protein hydrolysates with high biostimulating potential. The combined enzymatic–microbial approach showed a clear synergistic effect, promoting enhanced protein hydrolysis, which translated into superior nitrogen recovery from marine by-products. Agronomic assays under controlled conditions confirmed that these hydrolysates significantly improved wheat growth and biomass accumulation without impairing photosynthetic performance, with the combined treatment outperforming both individual processes at equivalent nitrogen levels.

Beyond their demonstrated agronomic effectiveness, the results highlight fertilizer production as a particularly suitable and sustainable end-use pathway for protein hydrolysates derived from food-processing residues. In contrast to their direct application as human nutritional ingredients, where stringent safety, sensory, regulatory, and market constraints may limit large-scale adoption, fertilizers from fish by-products offer a more immediate, robust, and impactful route for valorization. The use of marine-derived protein hydrolysates as fertilizers aligns with circular bioeconomy principles by closing nutrient loops, reducing reliance on synthetic nitrogen fertilizers, and mitigating organic waste streams. This positions biofertilization as a pragmatic and environmentally sound alternative for the large-scale utilization of protein hydrolysates.

Future studies to expand and deepen the actual findings should include the elucidation of the following:

- the hydrolysates’ effects as a function of the DH to determine the effect of the peptide chain length on the biostimulant activity,
- the microbial fertilizer’s effect as a function of fermentation time to determine the effect of fermentation products and microbial count on the biostimulant activity,
- field-scale effects and validation to establish the mechanistic basis of plant response, and
- economic feasibility to quantify operational costs and profits.

The study would also require chemical profiling of the hydrolysates and fermentation products, along with a scaled-up evaluation.

## Data Availability

The raw data supporting the conclusions of this article will be made available by the authors, without undue reservation.
